# Erythrocyte reference values in Emirati people with and without α^+ ^thalassemia

**DOI:** 10.1186/1471-2326-11-1

**Published:** 2011-02-24

**Authors:** Srdjan Denic, Abdul-Kader Souid, Nicolaas Nagelkerke, Saad Showqi, Ghazala Balhaj

**Affiliations:** 1 Department of Internal Medicine, Faculty of Medicine and Health Sciences, UAE University, Al Ain, UAE; 2 Department of Pediatrics, Faculty of Medicine and Health Sciences, UAE University, Al Ain, UAE; 3 Department of Community Medicine, Faculty of Medicine and Health Sciences, UAE University, Al Ain, UAE; 4 Department of Pathology, Al Ain Hospital, Al Ain, UAE

## Abstract

**Background:**

Interpreting the erythroid lineage in populations with high frequency of α^+ ^thalassemia allele is challenging due to the high prevalence of α^+ ^thalassemia homozygotes. For such populations, separate reference values for normal and α^+ ^thalassemia homozygotes are needed.

**Methods:**

We studied the erythroid lineage in 1,079 citizens of United Arab Emirates (UAE). Subjects with abnormal hemoglobin (39), iron deficiency (136) or erroneous entries (8) were excluded. MCV distribution in the remaining individuals (896) was visibly bimodal. Statistical mixture analysis with Normix program was used to separate subpopulations with normal and small red cells. Hardy-Weinberg equation was used to estimate genotype frequencies.

**Results:**

MCV of 78.0 fl separated phenotype-derived normal homozygotes (715) from phenotype-derived α^+ ^thalassemia homozygotes (181). The erythrocyte indices were significantly different between the two groups (*p *< 0.0001). The overall prevalence of phenotype-derived α^+ ^thalassemia homozygotes (-α/-α) was 0.20 and markedly varied among tribes, 0 to 0.31 (Mean = 0.15). The frequency of phenotype-derived α^+ ^thalassemia allele was 0.44; when accounting for tribal population structure and inbreeding, the calculated frequency was 0.34. These values were very similar to those found in the same population by genotyping and other phenotyping methods. The erythrocyte reference values for phenotype-derived normal homozygotes in Emiratis closely overlapped with those for Caucasians and normal homozygotes defined by genotyping. The reference values for phenotype-derived α^+ ^thalassemia homozygotes in Emiratis also closely overlapped with those for α^+ ^thalassemia homozygotes defined by genotyping.

**Conclusion:**

In populations with frequent α^+ ^thalassemia mutations, two sets of erythrocyte reference values could be determined without genotyping.

## Background

The prevalence of α^+ ^thalassemia allele in Peninsular Arabs varies between 0.07 and 0.58 [1-5]. In UAE nationals, the frequency of α^+ ^thalassemia allele is reported to be between 0.30 and 0.37 [1,2]. In these populations, the most common mutations are deletional, involving one of the linked pair of α-globin genes. Consequently, the genotypes are heterozygous α^+ ^thalassemia (-α/αα), homozygous α^+ ^thalassemia (-α/-α) and normal genotype (αα/αα). In contrast, deletional mutations of both paired genes (-/αα), α^o ^thalassemia, are absent in Gulf Arabs [1,6].

Most α-globin gene mutations decrease the size of red cells and alter other indices [6-9]. α^+ ^Thalassemia homozygote is characterized by lower hemoglobin (Hb), lower mean corpuscular volume (MCV), lower mean corpuscular hemoglobin (MCH) and higher red blood cell (RBC) count. α^+ ^Thalassemia heterozygotes have red cell indices that are between the normal genotype and α^+ ^thalassemia homozygotes [7,8]. A high frequency of α^+ ^thalassemia allele in a population often causes microcytosis and misinterpretation of the blood counts. This frequently leads to unnecessary testing and increases health costs. Additionally, in such populations the mean values of red cell indices are expected to be decreased and the standard deviations to be increased, as is also apparent from the reference intervals of red cells indices in two such populations [10,11]. Therefore, it is inappropriate for populations with high frequency of α^+ ^thalassemia allele to use the "Western" erythroid standards, developed for people with little α^+ ^thalassemia. In populations with high frequency of α^+ ^thalassemia allele, the use of separate reference values for normal and α^+ ^thalassemia homozygotes, similar to those in use for different genders and age groups, is more appropriate.

The erythroid standards for populations with high frequency of α^+ ^thalassemia are best defined with genotyping [8]. However, for most of them this is expensive and technically challenging. Nonetheless, the same goal may well be achieved with a combination of red cell phenotyping and mixture analysis. The resulting reference values, if properly validated, are still more appropriate than currently used standards derived for genetically different populations. This study is conducted to establish red cell reference standards for Emirati population by phenotyping and to validate them through comparison with the results of other studies.

## Methods

### Setting and study population

Details on study subjects have been reported previously [12]. Briefly, data were collected from 1,079 native UAE citizens, ethnically Arab, 538 females, 539 males and two of unknown gender. The age (mean ± SD) was 24.3 ± 6.3 years (range, 11 - 69); only 5 individuals were younger than 15 years.

The UAE population is tribal (67 tribes as per the 1968 census) and endogamous, and has high frequencies of α- and β-globin gene mutations [1,2,13]. Consanguineous marriages, which increase the likelihood of homozygosis, are common [14]. The government mandates and fully funds a premarital screening program for UAE citizens. The main purpose of the program is to decrease the incidence of *β*-thalassemia and sickle cell disease and marriages are not officially recognized without screening. All study subjects were participants of this program between March and August 2007.

### Study variables

Blood was collected in EDTA-tubes. Complete blood counts were performed once on each subject, using the Cell-Dyn Sapphire (Abbot Diagnostics, USA) analyzer. The hospital laboratory subscribed to external quality control conducted by United Kingdom National External Quality Assessment Scheme and met the analytic standards. Hemoglobin analysis was performed using high-pressure liquid chromatography (Variant II, Biorad Co.). Genotyping was not performed in this program.

### Selection criteria

Subjects (39) with abnormal hemoglobin (hemoglobin A_2 _> 3.5% or presence of hemoglobin S, D or E) were excluded from analysis. As iron deficiency was relatively common among UAE females and iron measurements were not routinely performed in this program, RDW ≥ 14.0 was used to exclude subjects (136) with iron deficiency. This cutoff was the upper limit (mean+2SD) for our male subjects (see Results). In the absence of iron deficiency, MCV differences between males and females were not statistically significantly different, corroborating the assumption of equivalency of their red cell sizes [7,8,15].

### Analytics

Erroneous or missing data were excluded: 13 RDW, five RBC counts, two MCV, six MCH and one hemoglobin A_2_. The study subjects were grouped and compared by gender and tribe, as identified by their last name. Standard descriptive and analytic statistical methods, such as histograms, linear regression and independent samples t-test were used.

**Figure 1:**
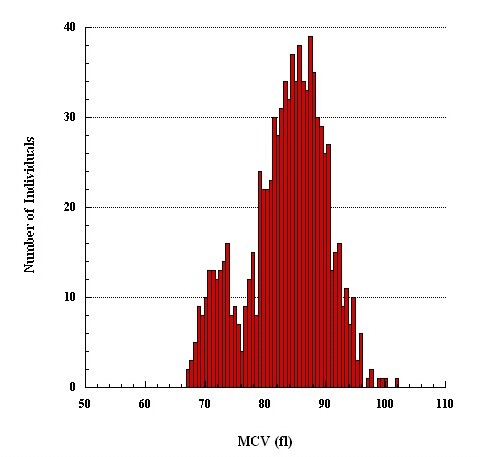
**Frequency distribution of MCV**.

As the distribution of MCV was visibly bimodal, a statistical mixture analysis was performed using PC-Normix program http://www.alumni.caltech.edu/~wolfe/normix.htm[16]. This analysis can identify two or more clusters with normal (Gaussian) distributions within a mixed population. In this study, the distribution of MCV appeared to comprise only two clusters (Figure 1). Further analysis of red cell parameters in the two populations (clusters) was performed using SPSS for Windows, Version 17.1. The allele frequency was derived from the frequency of low-MCV phenotype which was assumed to represent α^+ ^thalassemia homozygote. These calculations were performed for whole population and for each of the ten tribes. The phenotype-derived genotype frequencies were then calculated using Hardy-Weinberg formula corrected for inbreeding,. viz. for α^+ ^thalassemia homozygotes q^2^(1 - F) + qF, for α^+ ^thalassemia heterozygotes 2pq(1 - F) and normal homozygotes p^2^(1 - F) + pF [17]. The mean coefficient of inbreeding (*F*) in this population was previously found to be 0.022 (14), which we assumed to apply. The level of significance was set at <0.05.

### Ethical approval

The study was approved by Al Ain Medical District Human Research Ethics Committee.

## Results

**Figure 2:**
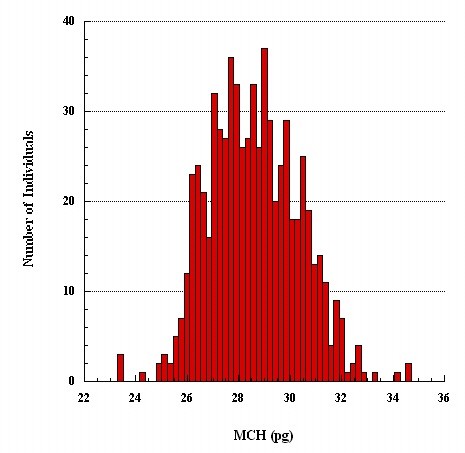
**Frequency distribution of MCH**.

The erythrocyte indices of 1,040 subjects without hemoglobinopathies other than α^+ ^thalassemia are shown in Table 1A. Subjects with RDW ≥14.0% were presumed to have iron deficiency (Table 2) and were excluded from estimating the reference intervals; an additional 8 subjects with incomplete data were also excluded. Their exclusion however only changed the values in the females (Table 1B). The frequency distribution of MCV in the remaining 896 subjects (Table 1B) showed two distinct subpopulations, but bimodal distribution of MCH was less distinct (Figure 1 and 2); the distributions of hemoglobin and hematocrit were apparently homogenous. Thus MCV was chosen to separate phenotypically normal from small red cells.

**Table 1:** Erythrocyte indices in the study subjects (A^a^) and in those with RDW <14.0% (B^b^)

			RBC(×10^6^/μL)	Hb (g/dL)	Hct (%)	MCV (fL)	MCH (pg)	MCHC(%)	RDW (%)
			Mean	Mean	Mean	Mean	Mean	Mean	Mean
		N	± 2SD	± 2SD	± 2SD	± 2SD	± 2SD	± 2SD	± 2SD
**A**	All	1,040	5.3	14	42.9	82.1	26.8	32.6	12.6
			4.0-6.5	10.5-17.5	33.2-52.7	66.6-97.6	20.6-33.0	30.4-34.8	9.2-15.9
	M	520	5.6	15.3	46.5	83.6	27.5	32.8	12.1
			4.5-6.7	13.0-17.6	40.5-53.0	70.1-97.1	22.0-33.0	30.7-35.0	10.3-13.9
	F	520	4.9	12.8	39.4	80.6	26.1	32.4	13.1
			4.0-5.9	10.2-15.4	32.5-46.2	63.8-97.3	19.5-32.7	30.2-34.6	9.0-17.2

**B**	All	896	5.3	14.4	43.8	83.4	27.3	32.3	12.1
			4.1-6.5	11.4-17.3	35.4-52.2	70.2-96.7	22.0-32.7	30.7-34.8	10.5-13.7
	M	495	5.6	15.3	46.6	83.8	27.6	32.8	12.0
			4.5-6.7	13.1-17.4	40.4-52.8	70.2-96.7	22.2-32.9	30.7-35.0	10.6-13.4
	F	401	4.9	13.2	40.4	82.9	27.1	32.6	12.2
			4.0-5.8	11.3-15.1	35.2-45.6	69.6-96.3	21.7-32.5	30.7-34.6	10.5-14.0

^a^Subjects with abnormal hemoglobin electrophoresis (n = 39) were excluded.

^b^Subjects with RDW >14.0% (n = 136, Table 2) were presumed to have iron deficiency and were excluded from estimating the reference intervals. Erroneous or incomplete data (n = 8) were also exclude from estimating the reference intervals.

**Table 2:** Erythrocyte indices of subjects with RDW > 14.0%.

		RBC(×10^6^/μL)	Hb (g/dL)	Hct (%)	MCV (fL)	MCH (pg)	MCHC(%)	RDW (%)
		Mean	Mean	Mean	Mean	Mean	Mean	Mean
	N	± 2SD	± 2SD	± 2SD	± 2SD	± 2SD	± 2SD	± 2SD
All	136	5.1	11.6	36.7	72.1	22.8	31.5	16.1
		3.8-6.4	8.1-15.0	26.9-46.5	57.1-87.0	16.9-28.7	29.2-33.9	11.7-20.4
M	14	5.9	14.0	44.2	75.2	23.8	31.6	15.2
		4.1-7.8	9.1-18.9	30.4-58.1	59.0-91.4	17.7-29.9	29.3-33.9	12.4-18.0
F	122	5.0	11.3	35.7	71.7	22.6	31.5	16.2
		4.0-6.0	8.6-13.9	28.5-43.0	57.0-86.3	16.8-28.5	29.1-33.9	11.7-20.6

Mixture analysis of MCV of 896 subjects found78.0 fl to best separate phenotypically normal from small red cells in the sense that the estimated probability of being phenotypically normal exceeded 0.50 for values ≥78 fl. Using a P-P (probability) plot, the empirical cumulative probability distribution of our MCV data plotted against that obtained by mixture analysis yielded an almost perfect straight, diagonal, line, suggesting an excellent fit. Consequently, reference intervals for subjects with normal phenotypes and α^+ ^thalassemia homozygous phenotype were based on the estimated means and standard deviations of the two constituent normal distributions of the mixture distribution and are shown in Table 3.

**Table 3:** Erythrocyte indices in subjects with normal (A) and small (B) red cells^a^

			RBC(×10^6^/μL)	Hb (g/dL)	Hct (%)	MCV (fL)	MCH (pg)	MCHC(%)	RDW (%)
			Mean	Mean	Mean	Mean	Mean	Mean	Mean
		N	± 2SD	± 2SD	± 2SD	± 2SD	± 2SD	± 2SD	± 2SD
**A**	All	715	5.1	14.5	44.1	86.1	28.4	33.0	11.9
			4.1-6.2	11.7-17.4	35.9-52.3	77.5-94.6	24.7-32.1	31.1-34.9	10.5-13.3
	M	398	5.4	15.4	46.8	86.4	28.6	33.1	11.8
			4.5-6.3	13.4-17.5	40.5-53.0	78.2-95.6	25.1-32.1	31.1-35.0	10.6-13.0
	F	317	4.8	13.4	40.8	85.6	28.1	32.9	12.0
			4.1-5.5	11.7-15.1	35.9-45.7	76.6-94.6	24.3-31.9	31.1-34.6	10.4-13.7

**B**	All	181	5.9*	13.6*	42.6*	73.0*	23.2*	31.9*	12.8*
			4.6-7.1	10.7-16.4	33.8-51.4	67.2-78.7	20.8-25.7	30.2-33.5	11.4-14.1
	M	97	6.3	14.6	45.8	73.1	23.3	31.9	12.7
			5.4-7.1	12.8-16.4	40.1-51.6	67.2-79.0	20.9-25.7	30.2-33.5	11.4-14.1
	F	84	5.4	12.4	39.0	72.8	23.2	31.8	12.9
			4.5-6.2	10.6-14.2	33.7-44.2	67.1-78.4	20.6-25.7	30.2-33.4	11.6-14.3

^a^MCV = 78.0 fl used as a separating value.

* P < 0.0001

**Figure 3:**
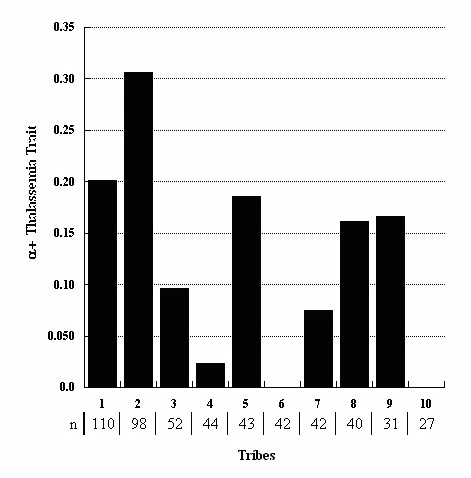
**Prevalence of phenotype-derived α^+ ^thalassemia homozygotes in ten largest tribes**.

When we treated the population as homogeneous, the estimated prevalence of phenotype-derived α^+ ^thalassemia homozygotes (181 of 896) was 0.20 and the prevalences of phenotype-derived α^+ ^thalassemia heterozygotes and normal homozygotes were 0.49 and 0.31, respectively; the estimated frequency of phenotype-derived α^+ ^thalassemia allele was 0.44. However, the prevalence of phenotype-derived α^+ ^thalassemia homozygotes varied substantially among the ten largest tribes (mean = 0.15), potentially vitiating the assumption of a single homogeneous population (Figure 3). Using stratification by tribe, the prevalence of phenotype-derived α^+ ^thalassemia heterozygotes was 0.45 and that of normal phenotype 0.40. Phenotype-derived α^+ ^thalassemia allele frequency in each tribe was adjusted for inbreeding and found to vary from 0 to 0.55. The aggregate frequency of the allele adjusted for population structure and inbreeding was 0.34.

## Discussion

**Figure 4:**
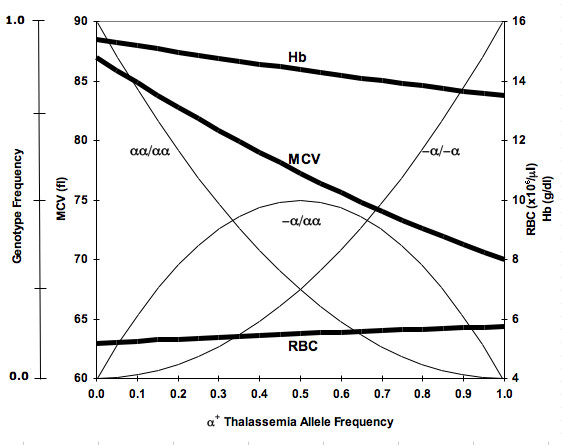
**Mean Hb, MCV and RBC values (thick lines) at different α^+ ^thalassemia allele frequencies**. Genotype frequencies (thin lines) are of αα/αα (normal homozygote), -α/αα (α^+ ^thalassemia heterozygote) and -α/-α (α^+ ^thalassemia homozygote). Data were created using Hardy-Weinberg equation and published values for the three genotypes [8,18].

The red cell reference intervals for male and female Emiratis are noticeably broader (Table 1) than in other Caucasian populations in which α^+ ^thalassemia is rare [15]. Similar observations were reported on young adults in Saudi Arabia, in whom the frequency of α^+ ^thalassemia allele varies between 0.07 and 0.5, as well as in Palestinians [10,11]. This finding is expected in any population with considerable variations in the number and size of red cells, i.e., α^+ ^thalassemia heterozygotes and homozygotes and normal homozygotes. In general, the effect of α^+ ^thalassemia allele frequency on the three genotypes and their aggregate effects on the mean values of red cell parameters are shown in Figure 4. This analysis shows that the standards developed in populations with frequent α^+ ^thalassemia are shifted to one side and wider, less precise, and critically depend on the frequency of α^+ ^thalassemia allele. Therefore, such populations require separate reference intervals, one for subjects with phenotypically normal red cells and another for those with small red cells.

We separated two cell populations based on their size (MCV) rather than MCH, despite earlier reports that MCH is more useful in separating α^+ ^thalassemia homozygotes from normal homozygotes [8]. The main reason for preferring MCV over MCH was that in our sample the frequency distribution of MCV was more clearly bimodal than that of MCH (Figures 1 and 2). As we were using mixture analysis (which breaks down a population into its constituent subpopulations by decomposing a frequency distribution into a mix of two normal distributions; here belonging to the normal and α^+ ^thalassemia homozygote phenotype subpopulations, respectively), MCV rather than MCH appeared a better parameter to accomplish this task. In the study that found MCH a more useful than MCV in separating normal from α^+ ^thalassemia homozygotes, the subjects' genotypes were known and α^+ ^thalassemia heterozygotes were excluded from analysis [8], which we could not do. Nonetheless, the distributions of normal and small red cell populations clearly overlap (Figure 1). In our study, a value of 78.0 fl seems to best separate subjects with normal red cells from those with small red cells. The validity of this finding is supported by the finding in another study in which the same value of MCV best predicted α^+ ^thalassemia homozygote defined by genotyping [18].

When tribal population stratification was taken into account (Figure 3) and adjusted for inbreeding, phenotype-derived α^+ ^thalassemia allele frequency was estimated at 0.34, and. the prevalence of phenotype-derived α^+ ^thalassemia homozygotes at 0.12. These results are similar to reports of homozygote frequencies obtained using genotyping (0.11) and other phenotyping (0.14) methods on the same population [1,2]. Heterogeneity in allele frequency among tribes may well be due to founder effects, with random numbers of α^+ ^thalassemia alleles segregated into subpopulations at the time of the foundation of current tribes. These differences were preserved by the practice of endogamy, which limits gene exchanges between the tribes. This substructure in the Emirati population (the consequence of tribal history) is also present in other Gulf Arab societies, and may explain reported variations in α^+ ^thalassemia frequency among different Arab populations.

Remarkably, nearly half of the studied population is deduced to be α^+ ^thalassemia heterozygous. Although in clinical practice these individuals are indistinguishable from normal, their erythroid indices are between the normal and α^+ ^thalassemia homozygotes [7,8]. Yet, contrary to expectations, the high prevalence of these phenotype-derived heterozygotes did not "blur" the bimodality of the distribution of MCV (Figure 1), suggesting that MCV values of most phenotype-derived α^+ ^thalassemia heterozygotes are well within the normal range. Indeed, in another study of red cell sizes in known genotypes, 64% of the α^+ ^thalassemia heterozygotes had MCV >78.0 fl [18].

**Figure 5:**
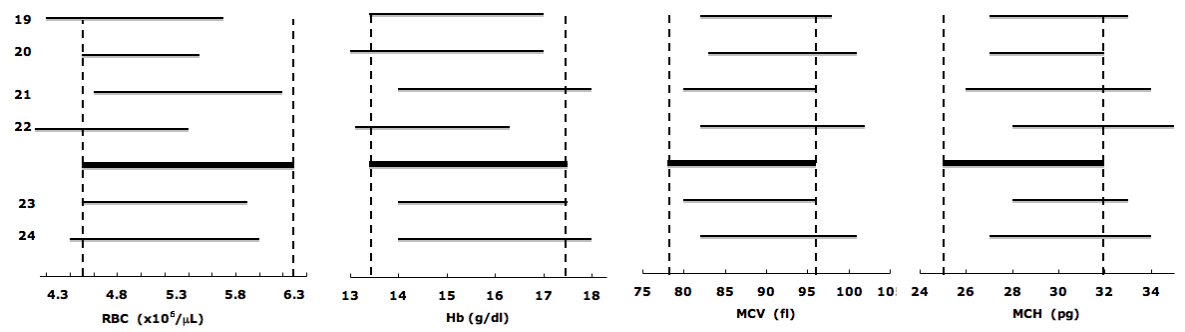
**Erythrocyte reference intervals for phenotypically normal Emiratis (thick lines, data from Table 3A) and Caucasians (thin lines)**. The RBC and Hb values are in males and the MCV and MCH values are in both genders. The numbers indicate references.

**Figure 6:**
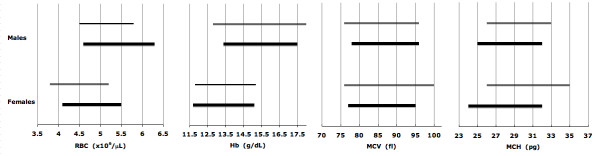
**Erythrocyte reference intervals for phenotypically normal Emiratis (thick lines, data from Table 3A) and Spanish population with αα^/^αα genotype (thin lines, data from Reference **[7]).

**Figure 7:**
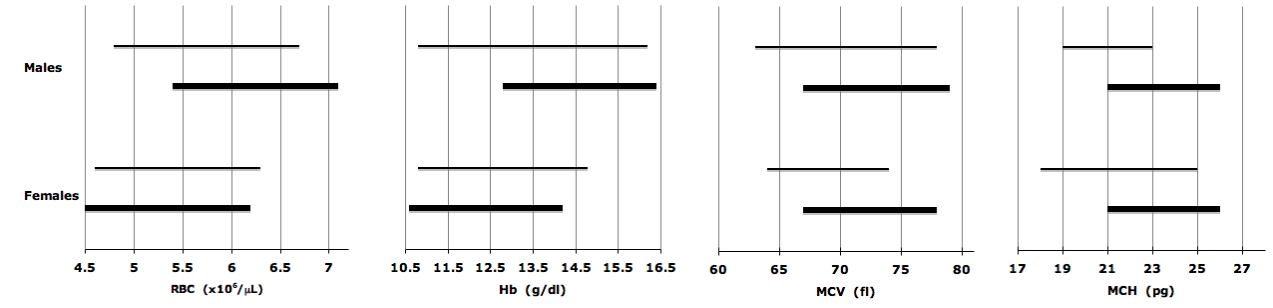
**Erythrocyte reference intervals for Emiratis with phenotype-derived α^+ ^thalassemia homozygosis (thick lines, data from Table 3B) and Spanish population with -α/-α genotype (thin lines, data from Reference**[ 7]).

As expected, the erythroid parameters for phenotype-derived normal and phenotype-derived α^+ ^thalassemia homozygotes are significantly different (Table 3). For phenotypically normal subjects, the reference intervals closely overlap with those for Caucasians in which α^+ ^thalassemia homozygosis is rare (Figure 5) [19-24]. Additionally, reference intervals markedly or completely overlap with the intervals published for adults genotyped as αα/αα (Figure 6) [7]. The results show that phenotypically normal Arabs have the same erythroid parameters as people of European origin. For phenotype-derived α^+ ^thalassemia homozygotes, the reference intervals overlap with those for adults with -α/-α genotype (Figure 7) [7]. These comparisons validate our results obtained with phenotyping and mixture analysis of phenotypes. The observed variations of reference intervals in Figures 5, 6, 7 and in other studies would seem attributable to sample size, subject selection, and sample handling and processing.

A possibly contentious issue is the use of RDW ≥14.0% to identify iron deficiency. This unsatisfactory test may have introduced errors in estimating the prevalence of phenotype-derived α^+ ^thalassemia homozygotes. This bias however is likely to be small, as nine times more women than men are excluded (Table 2), and the prevalence of phenotype-derived α^+ ^thalassemia homozygotes in two genders is not significantly different (*p *= 0.34). In general, in the absence of significant iron deficiency, which is more prevalent in women than in men, there is no evidence that MCV of men and women are different. Thus, the use of the upper limit of normal RDW in males (Table 1A) to exclude iron deficiency in the females seems reasonable. A similar value for the upper limit of normality of RDW is found in Caucasian males of comparable age [15].

## Conclusion

For clinical purposes, two sets of erythroid intervals are needed for populations with a high frequency of α^+ ^thalassemia allele. This study is the first to propose such reference intervals for clinical use, one for a population with normal and another for a population with small red cells (Table 3). The studied population is heterogeneous as regards the α^+ ^thalassemia allele, a heterogeneity that differs by tribal alliance. This study demonstrates a new approach for the development of red cell reference standards through a combination of phenotyping and mixture analysis. The reference interval it produced appear to be consistent with those obtained by genotyping. In addition, α^+ ^thalassemia allele frequency estimates using this method are similar to those obtained by genotyping. Thus both results support the validity of this approach. Our results are applicable to other Gulf Arabs of the same origin, i.e. old Bedouin. Also, our methods can easily be utilized in other populations with a high frequency of α^+ ^thalassemia homozygotes in which genotyping is not feasible or affordable.

## Competing interests

The authors report no conflict of interest. The authors alone are responsible for the content and writing of this article.

## Authors' contributions

SD conceived, designed and organized the study; furthermore, analyzed the results and wrote the manuscript. AKS conceived, designed the study, analyzed the results and wrote the manuscript. NN conceived and performed the statistical analysis and helped with the draft of the manuscript. SS participated in the design of the study, performed the tests and data collection. GB participated in analysis of the study. All authors read and approved the final manuscript.

## Pre-publication history

The pre-publication history for this paper can be accessed here:

http://www.biomedcentral.com/1471-2326/11/1/prepub
